# Construction of talent training mechanism for innovation and entrepreneurship education in colleges and universities based on data fusion algorithm

**DOI:** 10.3389/fpsyg.2022.968023

**Published:** 2022-09-23

**Authors:** Yuanbing Liu

**Affiliations:** Pinghu Normal School, Jiaxing University, Jiaxing, China

**Keywords:** data fusion algorithm, innovation and entrepreneurship education, talent training mechanism, construction research, information fusion

## Abstract

Nowadays, innovation and entrepreneurship courses occupy a very important place in universities and colleges and have also become an important teaching position in the process of building a new science. Colleges and universities actively respond to the challenge of “mass entrepreneurship and innovation” and define the goals and specifications of the talent training mechanism based on data fusion algorithms to cultivate as much high-quality applied talent as possible. In view of some shortcomings and problems in the current talent training mechanism in universities and colleges, this paper proposes a data fusion algorithm based on information fusion theory and proof theory. The aim is to verify the feasibility of establishing a talent training mechanism for innovation and entrepreneurship education in universities and colleges. And this paper analyzes and explores the data fusion algorithm and the elements of innovation and entrepreneurial talent training, and forms an operating mechanism for entrepreneurial talent training according to social needs. Among them, the efficiency of the data fusion algorithm used by the GM(1,1) model plays a significant role in the final result, and the minimum relative error value is 3.2%. Finally, it is concluded that we should focus on establishing a perfect talent training system for college students’ innovation and entrepreneurship education to improve students’ own comprehensive quality and various abilities, and to solve some social problems that are difficult to find employment in essence.

## Introduction

To adapt to modernization, colleges and universities need to offer training in innovation and entrepreneurship. In this societal context, one of the most important tasks for colleges and universities is to create a large talent pool in applied innovation and entrepreneurship. Colleges and universities need to update their concepts, identify their positioning in the education industry, continuously optimize teaching content and professional settings, strengthen the training of innovative teachers, and effectively strengthen their big data concepts. The concept of “data fusion” first appeared in the 1970s, and by the 1980s, the concept had become a professional discipline technology. The term “data fusion algorithm” is mostly used in cutting-edge technology research between scientific research institutes or enterprises, and has also been applied to the field of higher education over the years. It is a technology that integrates multiple disciplines such as signal processing, statistical estimation, pattern recognition, theory of operations, and artificial intelligence. Under normal circumstances, data fusion technology adopts information processing technology (Jardim, 2021). Within a complete algorithm framework, it automatically matches, associates, integrates and extracts features from multiple collected information, thereby completing the process of final evaluation and decision-making on the target object. The data fusion technology itself has been well developed, but how to apply it in the context of the talent training mechanism is a new issue worth thinking about.

In this paper, colleges and universities are the target of the research, and the main research method is the synthesis of data, which relates to the construction of the mechanism for training innovation and entrepreneurship talent in colleges and universities. This paper can attract more attention and establish a wider exchange and provide reference data on theoretical and practical research in this field for all countries.

Above all, it contributes to the progress and rapid development of data fusion algorithm technology and can be effectively applied to other fields. Humanity has entered the era of intelligence and information. The use and development of new technologies such as cloud storage, the Internet of Things, big data and artificial intelligence have profoundly changed production and human life, especially the way of thinking and learning.

Second, it contributes to the reform of higher education institutions and to improving the overall quality of students. For higher education institutions and universities to achieve the sustainable development goals, they must become more vital. At the core is the use of proactive and effective measures and technologies to deepen higher education reform and improve the quality of staff training to align with the strategy of developing innovation and entrepreneurship and ultimately serve social and economic development. Based on the research and analysis of the theories of various scholars, this paper combines the concept of data fusion algorithm from a holistic and system perspective. It comprehensively analyzes the mechanism of cultivating innovative and entrepreneurial talents in universities and colleges from several different perspectives and combines a variety of innovative research methods.

First, the study of theoretical innovation. Based on the theory of data fusion algorithm, this paper focuses on the construction of innovation and entrepreneurship education talent cultivation mechanism in colleges and universities and discusses the relationship between the two from different perspectives.

Second, research framework innovation. On the basis of relevant research, this paper boldly applies a new framework model, based on experiments, and on the basis of obtaining a large amount of data, effectively breaking through the limitations of previous theoretical research.

Third, research method innovation. After consulting a lot of data and literature, it is found that most of the research on this topic is based on quantitative research. This paper pays more attention to the integration of quantitative analysis and qualitative analysis, and uses different research methods to obtain widely applicable results.

Under the background of rapid social development, more outstanding talents with innovative and entrepreneurial abilities are needed to promote the progress of society, and to carry out professional innovation and entrepreneurship education for college students will enable college students to have a broader space for development and promote social progress improvement. Therefore, this paper adopts the algorithm of data fusion, combined with the education model of innovation and entrepreneurship in colleges and universities, to cultivate talents for contemporary college students.

## Related work

Yu X proposed a clustering-based adaptive predictive weighted data fusion algorithm. It establishes a prediction model based on the time correlation of the data, and adaptively adjusts the parameters of the prediction model by using the change trend of the early monitoring data, so as to predict the later data. The algorithm also calculates the reliability and weight of the monitored values and incorporates them (Yu et al., 2017).

To improve the survival time of the network, H. Wang wisely combines the BP neural network, genetic algorithm and particle swarm optimization algorithm and proposes an efficient intelligent data fusion algorithm GAPSOBP. Simulation results show that the GAPSOBP algorithm outperforms the LEACH and PSOBP algorithms (Wang et al., 2021) in terms of energy consumption and network lifetime. Zhao G proposes a data fusion algorithm based on fuzzy set theory and D-S proof theory, which addresses the shortcomings of existing solution-level algorithms for multidimensional data fusion. It determines the basic probability distribution of evidence based on fuzzy set theory and attribute weights and combines the data fusion of attribute evidence with the reliability of sensor nodes in the network (Zhao et al., 2020).

Nan DN is database-based and adopts six modes: Linear Discriminant Analysis (LDA), Quadratic Discriminant Analysis (QDA), k-Nearest Neighbors (kNN), Naive Bayes (NB), Classification Tree (CT), and Support Vector Machine (SVM). He uses recognition algorithms to classify single spectral and fused data. He proposed a set of data fusion mechanism based on intelligent data fusion and information fusion theory. In addition, he also analyzed that the size of the data will have a huge impact on the recognition accuracy of the data, which in turn will bring errors and mistakes to other branches and leaves of the entire data classification tree (Nan et al., 2020).

Yan Y. proposed measures to promote the development of student innovation and entrepreneurship in view of the current problems of student employment in higher vocational schools. He proposed a cooperation system between schools and enterprises and a joint talent development mechanism to transfer talent development from schools to enterprises and make full use of the advantages of enterprises in innovation and entrepreneurship training (Yan, 2018).

Qu Q. explores the problem of linking the talent training system in the maritime cultural industry and innovation cooperation between government, enterprises, universities, research institutions and users. It discusses how innovation can be incorporated into the maritime cultural industry talent training system, which is being jointly developed by the government, enterprises, universities, research institutions and users to overcome the barriers to human resource training and promote the dynamic development of the maritime cultural industry (Qu and Wei, 2018).

## Data fusion algorithm, innovation and entrepreneurship education in colleges and universities and talent training mechanism

Colleges and universities should actively use data fusion algorithms and innovation and entrepreneurship education models to guide students to participate in innovation and entrepreneurship in an orderly manner. In order to help students realize the importance of innovation and entrepreneurship for their own development, strengthen the education of students through data fusion algorithms, truly improve students’ innovation and entrepreneurship ability, and provide students with opportunities for innovation and entrepreneurship.

### Data fusion

**FIGURE 1:**
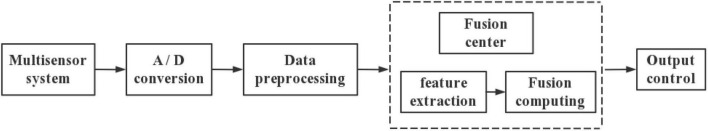
Data fusion process.

Data fusion is an information processing technology where computers are used to automatically analyze and integrate certain detection information from time series according to certain rules in order to perform necessary decision-making and evaluation tasks (Lee et al., 2017). The process includes basic signal acquisition, data preprocessing, feature extraction, fusion calculation and output of results. The process of data fusion is illustrated in Figure 1.

#### Data fusion model

**FIGURE 2:**
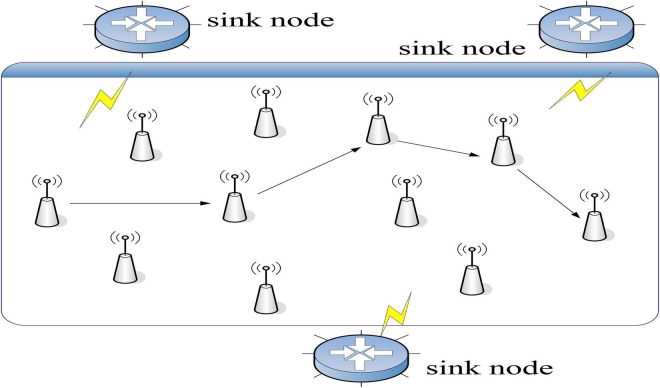
Schematic diagram of data aggregation transmission.

There are many classification methods for the construction of data models. From the perspective of information flow form and comprehensive processing level, they can be divided into four types: distributed, centralized, multi-level and hybrid (Donahue et al., 2017). The literature points out that the distributed data fusion model has the advantages of simple calculation, can add sockets, and each part can be processed separately, and is suitable for the talent training mechanism of innovation and entrepreneurship education in different universities. Firstly, the real-time data of colleges and universities are processed separately, and then data fusion is carried out. Through processing, the information obtained by each university has been corrected to a certain extent, and the reliability has been improved. Then, data fusion without feedback is carried out, which not only obtains relatively accurate information, but also improves the computing efficiency. The schematic diagram of data fusion transmission is shown in Figure 2.

#### Method of the project

In the research based on data fusion algorithm, this paper focuses on the data comparison and the correlation research between innovation and entrepreneurship education and talent training mechanism in colleges and universities, and makes preparations for this research purpose. In the writing of the thesis, the method of literature research, the method of fusion and intersection, and the method of combining theory with practice are used.

(1) Literary research methods: In the research process, research subjects acquire and collect relevant materials by reading literature, e-magazines and newspapers related to the development of creative skills and education in entrepreneurship. This article explores research and related study boundaries to gain a broader understanding and develop its perspective and classify the education system for creative skills and entrepreneurship education between colleges and universities. This article also provides a clear understanding of the current implementation of data integration algorithms through the introduction and comparative review of the relevant literature. This article examines and evaluates the fundamentals of the talent education system one by one and provides the basis for improving the structure of innovative education in innovation and entrepreneurship in colleges and universities.

**FIGURE 3:**
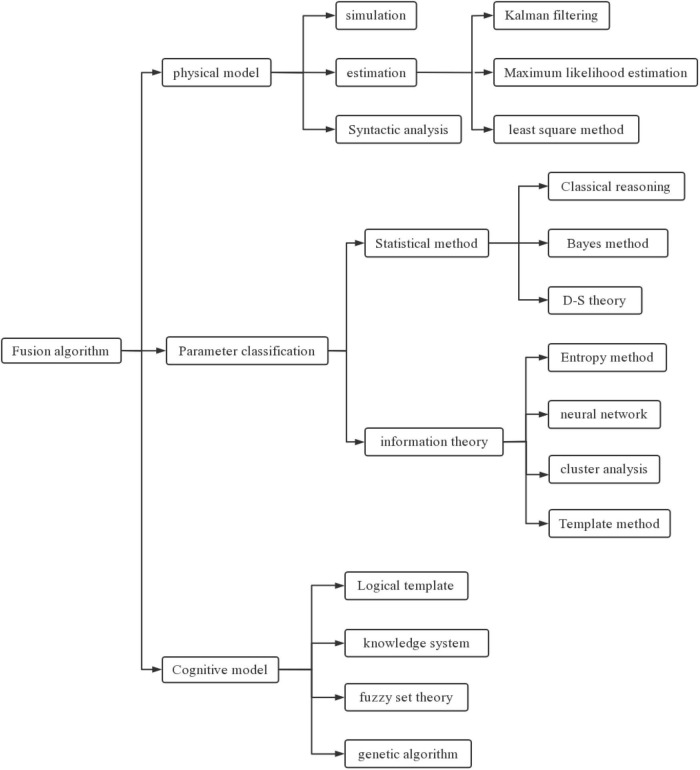
Conceptual classification of fusion algorithm.

There are many technologies and theories involved in data integration and there is currently no fully integrated algorithm that can adapt to any situation. Therefore, before using it is necessary to select a similar algorithm according to different application backgrounds (Newaz et al., 2018). According to the ideological classification of algorithms, they are divided into three main categories: physical models, variables, and intellectual models, as shown in Figure 3.

(2) Fusion cross method: The data fusion algorithm belongs to the science and engineering discipline. The research on the talent training mechanism of innovation and entrepreneurship education in colleges and universities often involves related disciplines such as management, psychology, talent science and education. This research uses the fusion and cross method to analyze and study the innovative talent training mechanism in colleges and universities under the background of multidisciplinary knowledge (Lu and Xu, 2018).

(3) Combining theory with practice: The construction of talent training mechanism itself is a social and humanistic activity, and the significance of all theoretical research needs to be proved by combining the actual situation in the end. It discusses how to integrate data analysis algorithms into the cultivation of innovative and entrepreneurial talents and play a good role in promoting and effective.

**FIGURE 4:**
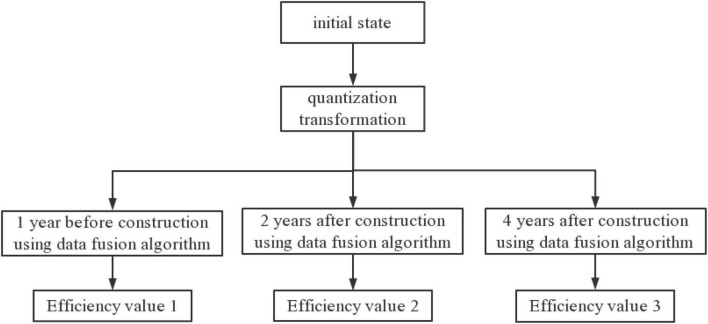
Flow chart of model algorithm based on data fusion algorithm optimization.

In this paper, quantitative compound transformation is used to change the initial state, and historical data and data fusion methods in different periods are used to establish a predictive analysis model, and the fusion analysis of different values is carried out to obtain the final optimization result. The flow chart of the optimization model is shown in Figure 4.

### Innovation and entrepreneurship education in colleges and universities

The main purpose of innovation and entrepreneurship education is to cultivate students’ innovative consciousness through certain extracurricular extension and expansion through learning professional theoretical knowledge and on the basis that professional courses meet the standard requirements. The target is usually aimed at students who are engaged in scientific research projects and students whose majors are groundbreaking (Hosseini et al., 2018).

#### Accurate positioning of educational philosophy

Innovation and entrepreneurship education in secondary and university education is a key factor in quality education and reflects a human-oriented educational philosophy. In the new era, as colleges and universities continue to innovate and educate and educate for entrepreneurship, the key is to change the traditional concepts of learning and introduce new scientific concepts mixed with study requirements. In the process of implementing various types of innovation and education and entrepreneurship management, colleges and universities should adopt a people-centered approach to education, consider the development of students’ modern professional skills, study and promote Full development of students. It should also organize community entrepreneurship training based on the development of innovative skills and then implement a science plan based on the reorganization of the university curriculum. In a well-developed and uniformed education framework in line with the individual development needs of students, establish and improve the education system of innovation and entrepreneurship, implement innovation and targeted education in entrepreneurship and increase the effectiveness of education. First of all, it is necessary to locate education on innovation and entrepreneurship in the secondary to integrate the concept of innovation and entrepreneurship education in all aspects of higher education and to integrate ideological and political science education and education. Vocational into higher education and universities… In the education of innovation and entrepreneurship, students in higher education and universities embrace the influence of entrepreneurship, encourage students to embrace entrepreneurship and participate in building creative and entrepreneurial communities. On the other hand, in line with the educational resources of higher education institutions and universities in different regions, the introduction of innovation and entrepreneurship education can be organically integrated with the development of higher education and global institutions. High school to improve primary education methods. And related practical relationships. Through the corresponding core courses in Innovation and Entrepreneurship and business education, it implements reforms and innovations appropriate to the education of innovation and entrepreneurship. It really creates a great environment for university students to learn about innovation and entrepreneurship and gives full play to the effectiveness of creative education and entrepreneurship.

#### Building an education incubation base

**FIGURE 5:**
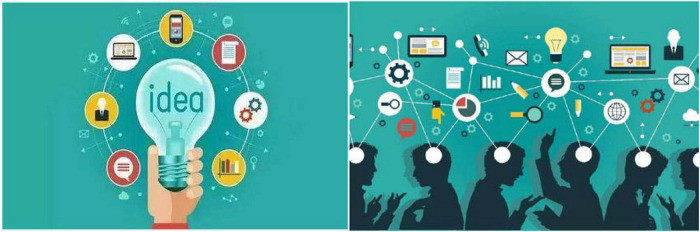
Demonstration of innovation and entrepreneurship education.

Education, innovation and entrepreneurship in secondary schools and universities are very useful and in a certain development process are important for creating an effective work environment. Through practical education, it can increase university students’ awareness of innovation and entrepreneurship and improve the overall quality of university students. The focus of education on innovation and entrepreneurship in higher education and colleges is student participation in entrepreneurship, and entrepreneurship should take the form of entrepreneurship (Cao et al., 2020). As a result of the “Number of Entrepreneurs and Innovations” policy, colleges and universities must not only educate students about entrepreneurship in education, but also focus on innovation in entrepreneurship education. First and foremost, higher education institutions and colleges should establish a training ground for innovation and entrepreneurship that combines the quality of higher education, supplementary planning, and entrepreneurship education to support entrepreneurship and employment. The second is to make full use of resources and organizations such as the University Business Association to promote self-management and self-innovation for university students. In addition, higher education institutions and universities will use comprehensive policies and systems to strengthen financial support for higher education institutions and universities, to strengthen the cooperation of activities between schools and companies, and to provide effective innovation and entrepreneurship monitoring services. Finally, create innovative out-of-campus and entrepreneurial education bases that provide students with effective vocational training opportunities, enable students to understand society as quickly as possible, and strengthen the impact of innovation and entrepreneurship education. Therefore, future colleges and universities should establish centers for innovation and entrepreneurship based on self-development. It incorporates the concept of innovation and entrepreneurship education through appropriate education, enhances general understanding of entrepreneurship and entrepreneurship and the ability of university students, and creates similar conditions for improving student entrepreneurial success. Figure 5 is an example of education in innovation and entrepreneurship in high schools and universities in new institutions.

#### Actively improve the curriculum teaching system

Innovative and entrepreneurial education in colleges and universities should fall into the category of quality education. It reflects an effective, economical, comprehensive and efficient curriculum system for innovation and entrepreneurship education. It combines unlike vocational training and combines innovation and entrepreneurship education in all areas. At this stage, when implementing the practical and educational activities of entrepreneurship, most colleges and universities often consider innovation and entrepreneurship education as part of traditional job counseling. Teaching about innovation and entrepreneurship is included in the teaching of job counseling, although it also has a similar effect on raising students’ awareness of innovation and entrepreneurship. However, in this teaching method, the curriculum for innovation and entrepreneurship education is limited and the lessons are limited, which does not affect good teaching (Liao et al., 2017; Zhang, 2019). Therefore, colleges and universities need to integrate advanced teaching concepts in the creative education system to be successful in creative education and entrepreneurship. In line with the needs of students at all levels of entrepreneurship education, it strengthens basic and vocational courses in innovation and entrepreneurship education and provides entrepreneurship training to all students to ensure that students receive Get a certain level of innovation and entrepreneurial education.. To create an effective creative and entrepreneurial classroom system for colleges and universities, it is also necessary to start with basic education and real struggle and organize courses such as basic unit teaching and entrepreneurship. An entrepreneurial park is set up on campus, allowing students to use entrepreneurial knowledge to carry out entrepreneurial competitions and complete entrepreneurial practice activities (Majumder et al., 2017). In this process, it can effectively improve students’ innovative and entrepreneurial spirit, give students more practical opportunities, and promote the accumulation of students’ entrepreneurial ability. After continuous practice and research, the entrepreneurial education model based on data fusion algorithm is a more feasible model. It can effectively combine innovation and entrepreneurship education with vocational education. Each major will open two graduation exits, and increase entrepreneurial exports on the basis of normal employment exports. After enrollment, students take entrepreneurship as their development goal, and carry out targeted innovation and entrepreneurship education combined with professional basic knowledge. In terms of curriculum system, entrepreneurship course is the main course to vigorously cultivate students’ entrepreneurial ability, management ability and innovation ability. It should be noted that the number of such classes should be strictly controlled, and the class personnel should be strictly selected. In the selection process, the written test and interview methods can be organically combined. It will select and organically gather students who have strong awareness, intention and ability of innovation and entrepreneurship in teaching practice and are suitable for entrepreneurship, and invite these students to participate in special education activities on innovation and entrepreneurship. It focuses on cultivating them, promoting them to grow into high-quality entrepreneurial talents that meet the needs of society, and improving teaching and practical effects. It cultivates more outstanding talents and provides corresponding human resource guarantee for socialist modernization (Hu et al., 2018; Xiuwu et al., 2018).

#### Improve students’ ability

External factors cannot be ignored, but the problems of college students themselves are more prominent. In the fierce competition, only by continuously learning and improving comprehensive skills can we gain a competitive advantage. First, college students should understand the current employment situation and make general forecasts of trends in the next few years to encourage themselves to value their time. It often pays attention to the employment dynamics of graduates, gives full play to its own advantages, understands the needs of the society for talents, and studies in a planned and purposeful manner. Secondly, for employed personnel, the ability to innovate and practice is the key. Especially in recent years, social enterprises pay more attention to the practical ability, work experience, learning ability and other factors of candidates while paying attention to academic qualifications. Therefore, college students should often participate in practical activities, broaden their horizons, and apply textbook knowledge into practice (Wan et al., 2018). For example, during the winter and summer vacations, people go to local companies for internships, familiarize themselves with the working environment and social rules, and accumulate practical experience. At the same time, they must dare to break through traditional thinking, be able to think independently, think about problems from different perspectives, and often put forward creative opinions. This requires continuous practice in daily life, such as participating in competitions such as college student innovation and entrepreneurship competitions during school, to encourage and guide students to participate in entrepreneurial practice. It provides students with opportunities for innovation and entrepreneurship, allowing students to accumulate entrepreneurial experience and lay a solid foundation for their future development (Bin et al., 2020; Son et al., 2020).

#### Construction education teachers

Innovation building and entrepreneurial teaching have a direct impact on the overall success of innovation and entrepreneurship education in colleges and universities. So you have to pay enough attention. It can usually start on all three sides. On the other hand, teachers in this field are actively encouraged to become entrepreneurs, increase job awareness and a deeper understanding of innovation and entrepreneurship. In addition to creative and entrepreneurial issues, it combines similar life events with teaching. In detail, it helps university students describe scientific entrepreneurship, combining innovation and entrepreneurship with education, and helps university students engage in innovation and entrepreneurship. The second is to encourage professors of creative education and entrepreneurship in colleges and universities to actively communicate and collaborate, create a good school spirit and actively implement projects such as expert training, workshops and internships. It will stimulate the teacher’s interest in transforming and improving the overall quality and quality of Teacher education. Finally, to establish a scientific assessment system for secondary school teachers and entrepreneurship, in addition to continuing the evaluation of traditional theoretical education, it is also necessary to select scientific evidence for teacher innovation and entrepreneurship education. There are detailed aspects of job guidance, entrepreneurship guidance, competition guidance, etc. And the content of innovation and entrepreneurship education is integrated into the teacher evaluation system. By evaluating progress to make construction more efficient, it creates a robust teacher system (Braconnot et al., 2018).

### Cultivation of “double creation” talents

There are many ways to build a talent training mechanism. In this paper, several common mechanism criteria are selected as the basic prediction methods, as shown in Table 1. The σ_θ_ /σ_*c*_ criterion has two grading criteria. Different grading criteria have different predictive effects on the mechanism (Deer et al., 2017). One of the purposes of this paper is to determine the degree of effectiveness of the mechanism at different stages.

**TABLE 1:** Some common criteria of algorithm.

Scholars	Index	Grading standard				Source literature
		No algorithm	Weak algorithm	Medium algorithm	Strong algorithm	
Yu Xiuwu	σ_θ_/σ_*c*_	<0.2	0.2∼0.3	0.3∼0.55	>0.55	Jardim, 2021
Haitao Wang	σ_θ_/σ_*c*_	<0.3	0.3∼0.5	0.5∼0.7	>0.7	Yu et al., 2017
Guangzhe Zhao	σ_*c*_/σ_*t*_	<0.5	0.5∼0.7	0.4∼0.6	<0.9	Wang et al., 2021
Di-Na Nan	*W*_ct_	<0.2	0.2∼0.3	0.3∼0.5	>0.5	Zhao et al., 2020
Yan Yang	Is	<0.5	0.5∼0.7	0.7∼0.9	>0.9	Nan et al., 2020

Generally speaking, the mechanism level determined by the criteria is a discrete variable (I, II, III, IV), and the mechanism implementation effect strength is a gradual transition process. Its category value is actually a continuous variable, and using continuous variables to describe the category of mechanism implementation effect strength can better reflect the actual situation. The use of continuous variable description mechanism to implement effect strength categories is also beneficial to the establishment of fusion prediction models.

## Data fusion prediction algorithm

**FIGURE 6:**
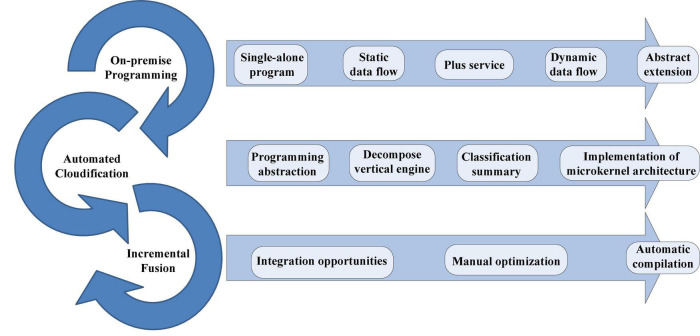
Thinking path of fusion computing.

For the multi-channel data reflected by various types of mechanisms measured by the system, the key step is to realize the data fusion processing. Data fusion technology is to intelligently analyze a large amount of data collected during the implementation of the mechanism, and according to a certain comprehensive analysis method, the measurement results can more accurately reflect the real situation, so as to realize the improvement function of the system (Saura et al., 2022). In order to use the fusion prediction method for mechanism construction, the data fusion prediction calculation model must first be established, that is, the fusion rules of each basic prediction method must be determined. Figure 6 shows the basic thinking path of data fusion computing.

The β value measured by m measurement data is weighted and averaged according to a certain weight, and the β value after data fusion can be obtained, which is recorded as $\bar{\beta }$, namely:


(1) $$\bar{\beta }=\sum_{i=1}^{m}w_{i}\,\beta_{i}=\left[w_{1},\ldots \,w_{n}\right]\,{\left[\beta_{1},\ldots ,\beta_{m}\right]}^{R}$$

Where *w*_*i*_ is the weight of the i-th mechanism. Since the random variable β_*i*_ obeys M(σ_*i*_^2^, θ_*i*_^2^), according to the multivariate probability distribution theory, the vector [β_1_ ⋯ β_*n*_] obeys the m-variable normal distribution M(b, A), and its distribution density function is as follows:


(2) qx=12π|m⁢/⁢2A|1⁢/⁢2exp {-12(y-b)A-1y-b)R}

Where:


(3) $$Y=\left[y_{1}\,\cdots \,y_{n}\right]$$

(4) $$b=\left[\theta_{1}\,\cdots \,\theta_{m}\right]$$

(5) $$A=\left[\beta_{1}\,\cdots \,\beta_{n}\right]\,\left\lfloor \sigma_{1}^{2},\ldots ,\sigma_{n}^{2}\right\rfloor$$

Then the distribution density function of the linear combination $\bar{\beta }$ of [β_1_ ⋯ β_*n*_] is:


(6) q⁢(x)=1(2π)(∑i=1mwi2σi2)1⁢/⁢2m⁢/⁢2⁢exp ⁡{-(x-∑i=1mwiμi)22⁢∑i=1mwi2⁢σi2}

Because of $\sum_{i=1}^{m}w_{i}=1$, the extreme value theory can be obtained from the constraints. To make $\bar{\sigma }$ the minimum value, the weights of the mechanism must meet the following conditions:


(7) $$w_{i}=\frac{1}{\sigma_{i}^{2}\,\sum_{i=1}^{m}\frac{1}{\sigma_{i}^{2}}}$$

At this point, the value of $\bar{\sigma }$ is:


(8) $${\bar{\sigma }}_{\min}\,\frac{1}{\sqrt{\sum_{i=1}^{m}\frac{1}{\sigma_{i}^{2}}}}$$

It can be seen that adding a mechanism involved in data measurement and fusion, regardless of the accuracy of the mechanism, the final fusion data accuracy must be higher than the data accuracy of any mechanism, namely:


(9) $${\bar{\sigma }}_{m}=\frac{1}{\sqrt{\sum_{i=1}^{m}\frac{1}{\sigma_{i}^{2}}}}=\frac{1}{\sqrt{\sum_{i=1}^{m-1}\frac{1}{\sigma_{i}^{2}}}+\frac{1}{\sigma_{m}^{2}}}\,\text{p}\,\frac{1}{\sum_{i=1}^{m-1}\frac{1}{\sigma_{i}^{2}}}={\bar{\sigma }}_{m-1}$$

Bayesian statistical processing methods are based not only on sample information expressed in existing data, but also on empirical information embodied in prior distributions. On the basis of estimation and verification, the statistical decision theory considering the after-effect is adopted (Dao et al., 2017). In the data processing part, the Bayes data estimation fusion algorithm is used, which greatly improves the accuracy and level of the construction effect of the talent training mechanism for innovation and entrepreneurship education in colleges and universities.

Let the parameter θ in the total distribution function X(x, θ) be a random variable, and the population is randomly sampled in order to obtain the existing information of θ, namely:


(10) $$x={\left(x_{1},x_{2},x_{3},\ldots ,x_{n}\right)}^{T}$$

Let the joint probability distribution density of sample x and parameter θ be q(x, θ), and the joint probability distribution density of parameter θ be q(x, θ). Then the conditional distribution density of x to θ is *q*_*x*_(*x*|θ), also known as the likelihood function, we can get:


(11) $$q\,\left(x,\theta \right)=q_{x}\,\left(x|\theta \right)\,q_{\prod }\,\left(\theta \right)=L\,\left(x,\theta \right)\,q_{\prod }\,\left(\theta \right)$$

Where q_*II*_(θ) is the prior distribution determined by the prior information of the parameter θ. From the conditional probability distribution formula, the formula can be obtained:


(12) $$q\,\left(x,\theta \right)=q_{x}\,\left(x|\theta \right)\,q_{\prod }\,\left(\theta \right)=q_{h}\,\left(\theta |x\right)\,q_{x}\,\left(x\right)$$

Then *q*_*h*_θ|*x* is the posterior distribution of the sample, that is:


(13) $$q_{h}\,\left(\theta |x\right)=q_{x}\,\left(x|\theta \right)\,q_{\prod }\,\left(\theta \right)\,/\,q_{x}\,\left(x\right)$$

Represented by the x-edge distribution of the joint distribution, the formula can be written as:


(14) $$q_{h}\,\left(\theta |x\right)=q_{x}\,\left(x|\theta \right)\,q_{\prod }\,\left(\theta \right)\,/\,\int q\,x\,\left(x|\theta \right)\,q_{\prod }\,\left(\theta \right)\,d\theta$$

Then the Bayesian estimate of θ is:


(15) $$\theta =E\,\left\{q_{h}\,\left(\theta |x\right)\right\}$$

A conventional linear fusion system model, its state space formula is:


(16) $$y_{n}=R_{n-1}\,y_{n-1}+H_{n-1}\,u_{n-1}+w_{n-1}$$

The detection formula is:


(17) $$x_{n}=G\,y_{n}+c_{n}$$

In this paper, a simple and practical linear weighted sum method is used to establish a data fusion prediction calculation model, and its mathematical expression is as follows:


(18) $$Z=\sum_{i=1}^{n}w_{i}\,Z_{i}$$

In the formula: Z is the predicted value of data fusion; *Z*_*i*_ is the predicted value of the i-th basic prediction method S_*i*_; *w*_*i*_ (i = 1,2,…n) is the weight of the i-th basic prediction method S_*i*_, and it satisfies:


(19) $$\sum_{i=1}^{n}w_{i}=1$$

Due to the limitations of different criteria, the predicted results may be different from the actual situation. Therefore, it is necessary to analyze the difference law of the prediction results of different criteria and evaluate the rationality of different criteria, so as to provide an objective basis for the weight calculation and ensure the reliability of the fusion prediction results. Therefore, based on the data fusion algorithm, this paper establishes the criterion rationality evaluation and weight calculation method, and uses the concept of support in the data fusion algorithm as the basis for weight calculation. The support degree represents the degree of mutual support between the two sets of data (Saura et al., 2021).

If the support degree between the predicted result and the actual result of a certain criterion is higher, it means that the criterion is more reliable, and a relatively larger weight should be assigned. Therefore, it is most reasonable to use the actual results as the reference object in the support calculation, and the weights obtained from this are more realistic. However, the actual situation of the algorithm is not clear, it is required to make predictions. Therefore, it is impossible to determine the weight of each model criterion through the actual results, and the weight can only be determined through the data itself, and the data fusion algorithm can just solve this problem. Under the premise that most of the criteria are reasonable, the overall data itself is used as the reference object. If the overall data supports the prediction result of a certain criterion, the more reliable the prediction result is, and the corresponding criterion should be given greater weight.

The formula for calculating support is usually as follows:


(20) $$r_{i\,j}=e^{-f_{i\,j}}$$

In the formula:


(21) $$f_{i\,j}=\left|x_{i}-x_{j}\right|$$

i,j = 1,2,…,m; *x*_*i*_ (i = 1,2,…,m) is a set of observations; *f*_*ij*_ represents the relative distance between *x*_*i*_ and *x*_*j*_. From formula (20), it can be seen that the larger the relative distance between *x*_*i*_ and *x*_*j*_, the smaller the support degree, and with the increase of the relative distance, the decreasing degree shows an increasing trend.

**FIGURE 7:**
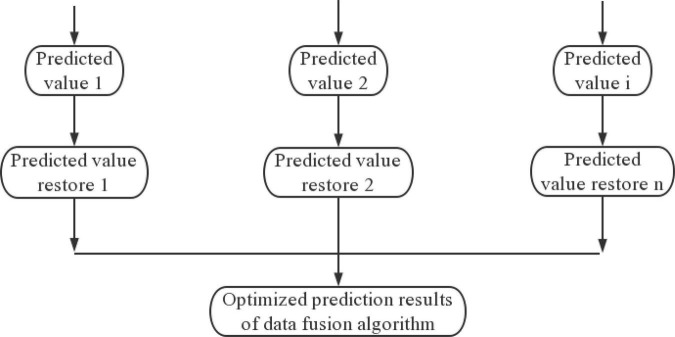
Flow chart of GM (1,1) model algorithm based on data fusion algorithm optimization.

By studying the construction model of the talent training mechanism for innovation and entrepreneurship education in colleges and universities, in this paper, the general GM (1,1) model (PGM) and the GM (1,1) model (OGM) optimized based on the data fusion algorithm proposed in this paper are used for simulation and prediction experiments. The experimental comparison process is shown in Figure 7.

Tables 2, 3 show the comparison experiments of the simulation and prediction results of the ordinary GM (1,1) model (PGM) and the data fusion algorithm optimization model (OGM).

**TABLE 2:** Result 1.

Raw data (unit:cm)	Prediction results of GM (1,1) model (PGM)	Prediction results of optimized data fusion model (OGM)
1.45	1.47	1.49
1.51	1.50	1.54
1.53	1.55	1.58

**TABLE 3:** Result 2.

Raw data (unit:cm)	Prediction results of GM (1,1) model (PGM)	Prediction results of optimized data fusion model (OGM)
1.52	1.59	1.62
1.56	1.62	1.65
1.58	1.65	1.71

Tables 2, 3 show that the minimum value of OGM’s increase is 0.02, and the maximum value is 0.13. The minimum value of the increase in PGM is 0.02, the maximum value is 0.07, and one of the data is down. Therefore, it is proved that the prediction result optimized by the data fusion algorithm is much better than the simulation prediction effect of the ordinary GM model, and the degree of improvement is obvious.

**FIGURE 8:**
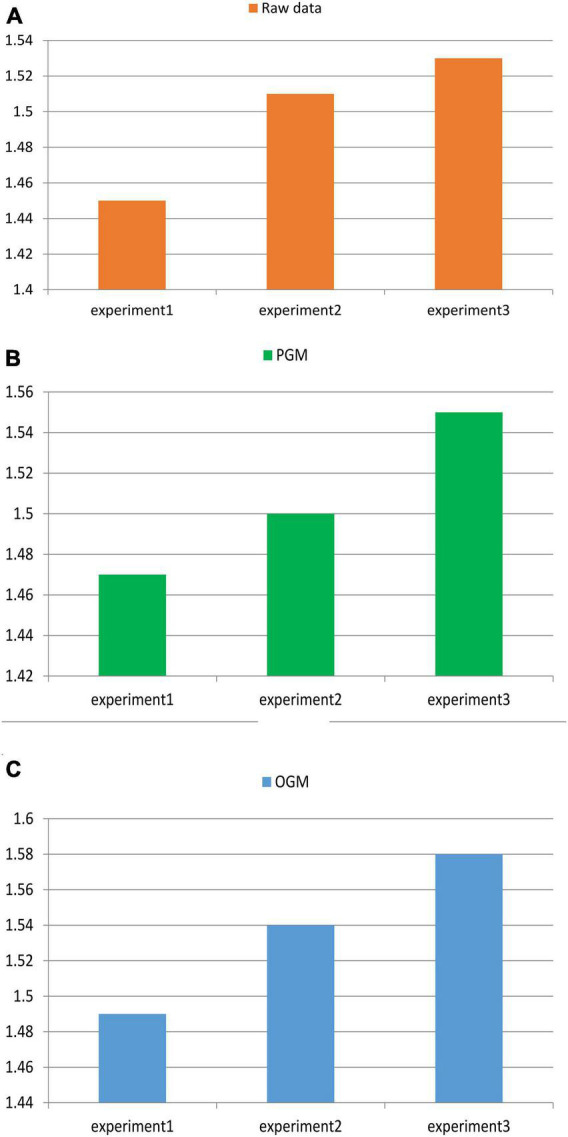
Comparison of results. **(A)** Raw data results in the experiment. **(B)** Experimental results of the PGM model. **(C)** Experimental results of the OGM model.

The comparison curve between the original data and the original data after compound transformation is shown in Figure 8.

Figure 8 shows that, in Experiment 1 and Experiment 3, the data has been on an upward trend; Experiment 2 is affected by a single original data, and the data shows the characteristics of instability. After the compound transformation, the data increased significantly, with a linear increase, an increase of 0.1 percentage points, and the effect was greatly improved.

**FIGURE 9:**
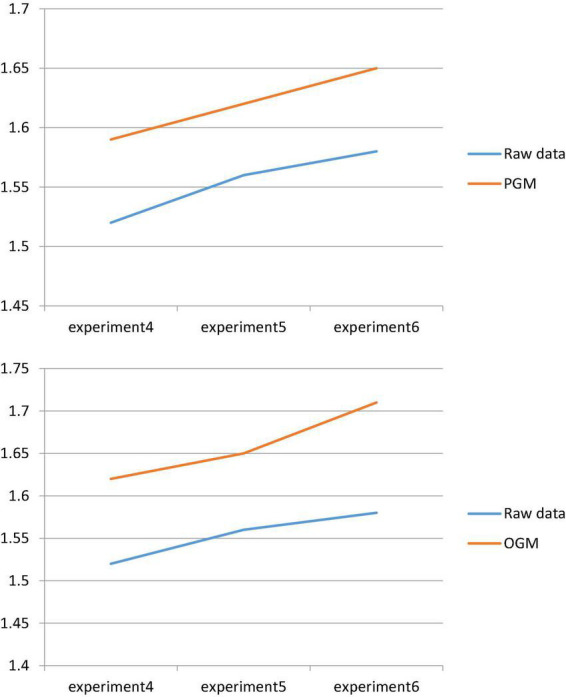
Comparison of results.

Figure 9 shows that the data optimized by the GM model and the fusion algorithm has a relatively large gap compared with the original data, and there is still a lot of room for improvement. Among them, the maximum increase rate of PGM is 0.07, and the minimum value is 0.06; the maximum increase rate of OGM is 0.13, and the minimum value is 0.09. This shows the superiority of OGM and PGM, with OGM slightly better than PGM.

Table 4 is a comparison of the detection results of the talent training mechanism based on data fusion.

**TABLE 4:** Comparison of fusion results.

Fusion algorithm	Fusion result value	Relative error/%
Optimal fusion algorithm	12.8	3.2
	13.1	4.8
Average algorithm	14.8	18.7
	15.0	20.0
Reliability algorithm	14.5	16.9
	14.7	17.6

It can be seen from Table 4 that the predicted value of the GM(1,1) model after data fusion optimization is more accurate than the predicted value of the GM(1,1) model, and the minimum error value is 3.2%.

**FIGURE 10:**
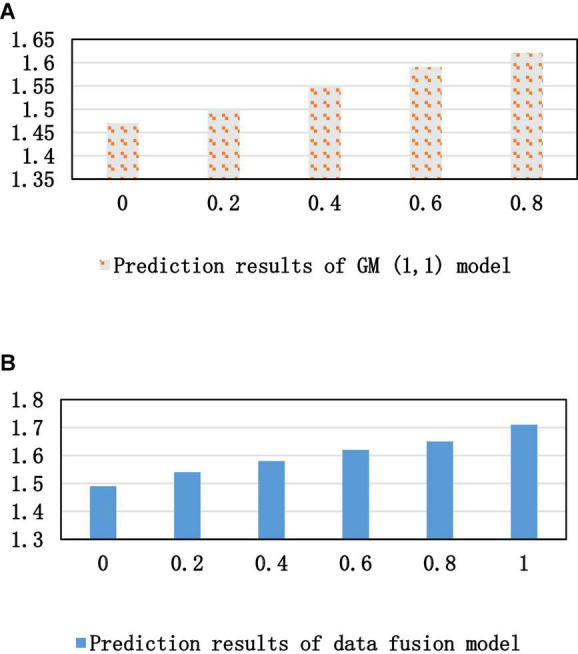
Prediction results of two models. **(A)** Prediction results of the GM model. **(B)** Prediction results of the data fusion model.

**FIGURE 11:**
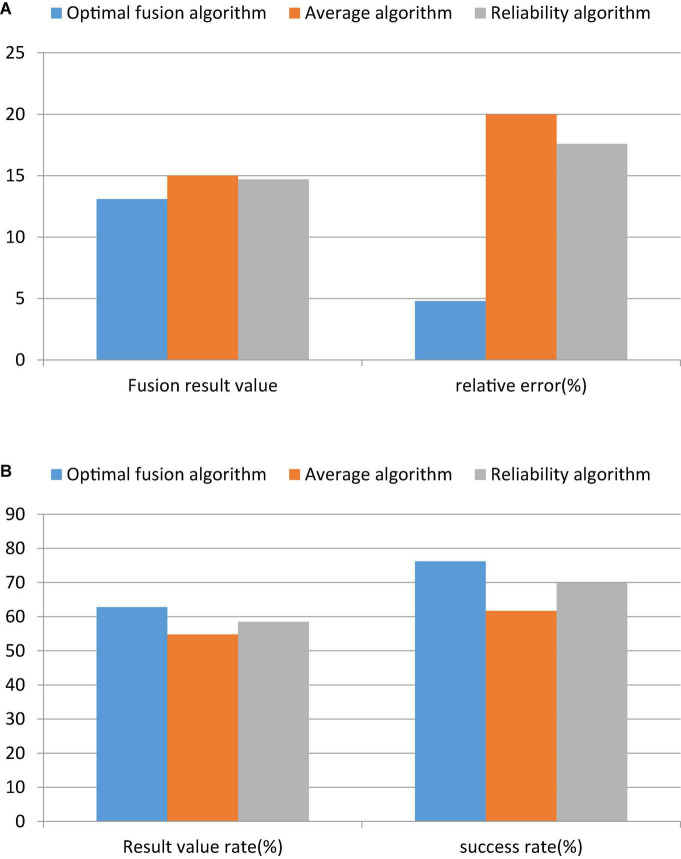
Comparison of results based on data fusion. **(A)** Data fusion algorithm calculation results before optimization. **(B)** Calculation results of the optimized data fusion algorithm.

Figure 10 shows that each group of data has been greatly increased after the optimization of the data fusion model, and the minimum value of the increase is 0.02 and the maximum value is 0.04. That is, as the prediction time increases, the prediction result graph of the optimized GM(1,1) model is closer to the real graph. Therefore, the optimization analysis model of data fusion is more worthy of reference in the construction, analysis and prediction of the talent training mechanism of innovation and entrepreneurship education in colleges and universities, as shown in Figure 11.

Figure 11 shows that the optimized fusion algorithm has the smallest error, which is as low as 4.8%, and the highest success rate, which is 76.2%. By using the GM(1,1) prediction model and the data fusion theory, the GM(1,1) data fusion prediction algorithm is obtained. Finally, the following conclusions are drawn based on the calculation results of the example.

(1) Compound transformation is an effective method to improve the prediction accuracy of GM(1,1) model. Using the GM(1,1) model for prediction, due to the continuous accumulation of its own errors, the long-term prediction results are more and more deviated from reality. Constant iteration of actual measurements and iso-dimensional reconstruction of the model can lead to constant self-correction of the model.
(2) Compared with the ordinary GM(1,1) model, the data fusion optimization results based on the GM(1,1) model use different amounts of raw data to simulate, predict and optimize multiple results. It effectively avoids the large deviation of the prediction results caused by the mutation of a single original data, maximizes the mining of the original data, and improves the reliability of the prediction results.
(3) The example shows that the GM(1,1) model based on data fusion optimization is worthy of reference in the construction and design of talent training mechanism. Through the data, the data fusion algorithm proposed in this paper effectively achieves the purpose of making full use of the obtained measured data to calculate the reliability and stability of the talent training mechanism.

## Discussion

This paper proposes to use data fusion algorithm to strengthen the innovation of talent training mechanism, and on this basis, conceives and preliminarily designs the research framework and method of talent training mechanism innovation. The expected function is finally achieved through the experimental test. This paper provides intellectual support for a more systematic and in-depth exploration of the innovative talent training mechanism. Innovating the talent training mechanism in colleges and universities is the top priority of comprehensively deepening the comprehensive reform of higher education. How to build a talent training mechanism is of great significance to the quality and connotation of talent training in colleges and universities. The “algorithm + mechanism” studied in this paper has certain practical application value and broad market prospects. Finally, the predicted value of the GM(1,1) model after data fusion optimization is more accurate than that of the GM(1,1) model, and the minimum error value is 3.2%. After the optimization of the data fusion model, each group of data has a great increase, the minimum value of the increase is 0.02, and the maximum value is 0.04. That is, as the prediction time increases, the prediction result graph of the optimized GM(1,1) model is closer to the real graph.

## Conclusion

“Entrepreneurship and innovation” are the “twins” of the new normal economic development. As a result, education, innovation and entrepreneurship have become hot topics in higher education, and the development of innovation and entrepreneurial skills has become a common goal of higher education institutions and universities. Universities and academies are actively seeking more honest, complete and appropriate approaches to innovation and entrepreneurship education. In fact, it has contributed to the development of the theory and practice of innovation and entrepreneurship education in colleges and universities, and has achieved some success and successful experiences. However, there are still many problems in creating an equitable and well-known scientific model for innovation and entrepreneurship education. It has faced difficulties from theoretical research to practice, and the educational model of talent has severely hampered the development of innovation and entrepreneurship education in colleges and universities. Therefore, it is urgent to find practical solutions and start on a new path of development in education, innovation, and entrepreneurship.

In the current society, due to changes in the social structure, the employment positions provided by all walks of life show a downward trend, but the number of college graduates continues to increase every year, which to a large extent leads to the need for college students after entering the society. face the problem of employment difficulties. In order to improve this social status quo, relevant management departments and the government should carry out education to promote innovation and entrepreneurship, and focus on establishing a sound talent training system for college students’ innovation and entrepreneurship education to improve students’ own comprehensive quality and various abilities, and to solve the social problem of employment difficulties in essence.

This study investigates and develops talent training models for innovation and entrepreneurship education based on knowledge integration algorithms. This thesis believes that this model is based on the treatment of all students and plays an important role in solving the current problems of innovation and entrepreneurship education in colleges and universities. A general understanding of the society of innovation and entrepreneurship and its importance is also being developed gradually. It is possible that education on innovation and entrepreneurship will be included in all links in the future education system.

## Theoretical implications

Through the analysis of the above problems, we are fully aware of the importance and necessity of colleges and universities to pay attention to the construction of a talent training system for college students’ innovation and entrepreneurship education.

## Practical implications

The development of data fusion technology and the integration of innovation and entrepreneurship in the new era with modern information technology have improved the quality of innovation education in colleges and universities, and will effectively promote the development of information technology and innovation and entrepreneurship education.

## Limitations and future research

However, it is undeniable that there are still various problems to be improved in the construction of the actual talent evaluation system, which are mainly reflected in the traditional and single means of innovation and entrepreneurship education, the lack of a perfect management mechanism and the lack of a modern practice platform. It requires teachers to look at the problems existing in the construction of the talent training system from a correct perspective, and focus on optimizing and updating the innovation and entrepreneurship education talent education system and training system for college students in the follow-up work, and organize a team of talents with strong professional skills. Cultivate a team of teachers, realize professional teaching work for college students, and lay a solid foundation for promoting the smooth development of teaching work and cultivating college students’ innovation and entrepreneurship ability.

## Data availability statement

The original contributions presented in this study are included in the article/supplementary material, further inquiries can be directed to the corresponding author.

## Author contributions

The author confirms being the sole contributor of this work and has approved it for publication.
